# *Leishmania amazonensis* Promastigotes or Extracellular Vesicles Modulate B-1 Cell Activation and Differentiation

**DOI:** 10.3389/fcimb.2020.573813

**Published:** 2020-10-30

**Authors:** Natasha Ferraz de Campos Reis, Talita Vieira Dupin, Carolina Rizzaro Costa, Maytê dos Santos Toledo, Vivian Cristina de Oliveira, Ana Flavia Popi, Ana Claudia Torrecilhas, Patricia Xander

**Affiliations:** ^1^ Laboratory of Cellular Immunology and Biochemistry of Fungi and Protozoa, Department of Pharmaceutical Sciences, Federal University of São Paulo, São Paulo, Brazil; ^2^ Department of Microbiology, Immunology and Parasitology, Paulista School of Medicine, Federal University of São Paulo, São Paulo, Brazil

**Keywords:** *Leishmania*, extracellular vesicles, B-1 cells, innate immune response, activation

## Abstract

B-1 cells are considered an innate-like B cell population that participates in effective innate and adaptive responses to pathogens. B-1 cells produce immunoglobulins, cytokines, chemokines, migrate to inflammatory sites, and differentiate into mononuclear phagocyte-like cells. Murine B-1 cells phagocytosed *Leishmania* *in vitro* and *in vivo* and participate in immunity against *Leishmania*. Our group showed that B-1 cells or their extracellular vesicles (EVs) led to a resistance to experimental infection by *L. amazonensis.* However, the B-1 cells’ responses to *Leishmania* or EVs isolated from parasites are still poorly characterized. Studying the activation and differentiation of B-1 cells *in vivo* can contribute to a better understanding of how these cells participate in immunity to *L. amazonensis.* Thus, we evaluated the expression of myeloid (M-csfr, G-csfr, Spi-1) and lymphoid (EBF, E2A, IL-7R) lineage commitment factors, Toll-like receptors (TLRs), activation cell surface markers, nitric oxide (NO) and reactive oxygen species (ROS) production in murine peritoneal B-1 cells collected after 24 or 48 h post-infection with *Leishmania (Leishmania) amazonensis* promastigotes or EVs released by the parasites. Our results demonstrated that *L. amazonensis* infection did not stimulate the expression of CD40, CD80, CD86, F4/80, and MHC II in B-1 cells, but a significant decrease in the production of NO and ROS was observed. The infection induced a significantly higher arginase expression in B-1 cells, but the stimulation with EVs led to a decrease in this gene expression. TLR-2 and TLR-6 had significantly higher expression in B-1 cells from mice intraperitoneally stimulated with the parasite. The TLR-9 expression was higher in animals infected or stimulated for 48 h with EVs. Interestingly, in B-1 cells the stimulus with *L. amazonensis* led to a substantial increase in the expression of myeloid restricted transcription factors. Thus, our study suggests that the parasites or EVs differently modulated the activation and differentiation of B-1 cells.

## Introduction

*Leishmania* release virulence factors in extracellular vesicles (EVs) that can interact with host cells, modulate host immune systems, contributing to the *Leishmania* infection (Silverman and Reiner, 2011; Atayde et al., 2016). Many groups have been studying the role of EVs in *Leishmania* infection, focusing on parasite-host cell interaction, and innate immune response (Santarém et al., 2013; Atayde et al., 2015; Atayde et al., 2019; Dong et al., 2019).

EVs are components secreted by mammalian cells (Théry et al., 2018), bacteria (Lee et al., 2016), fungi (Vallejo et al., 2012a; Vallejo et al., 2012b; Ikeda et al., 2018), and parasites (Torrecilhas et al., 2012; Marcilla et al., 2014; Campos et al., 2015; Evans-Osses et al., 2017; Ribeiro et al., 2018). They represent a new type of intercellular communication since they are composed of DNA, RNA, proteins, lipids, and cellular metabolites (Kalra et al., 2016; Théry et al., 2018). *In vitro*, EVs released by *Leishmania* modulated cytokine production, cell surface marker expression, and microbicidal molecule production by human and murine phagocytic cells (Silverman et al., 2010a; Silverman et al., 2010b). *In vivo*, experimental models of leishmaniasis have shown that the inoculation of the parasites in the presence of the EVs led to an increase in the lesion size and inflammation, supporting the hypothesis that EVs released by the *Leishmania* have a role in parasite infection (Atayde et al., 2015; Barbosa et al., 2018).

In tropical regions are an estimated 1.3 million new cases of leishmaniasis diagnosed annually with 20,000 to 30,000 deaths (Alvar et al., 2012; Burza et al., 2018). *Leishmania* spp. are protozoan parasites, etiologic agents of leishmaniasis, a debilitating, and often disabling disease (Burza et al., 2018). The clinical forms of leishmaniasis depend on the *Leishmania* species (Subramanian and Sarkar, 2018), vector characteristics (Rogers, 2012), and the host’s immune response (Kaye and Scott, 2011; Scott and Novais, 2016). The macrophages are the central cells in immunity against *Leishmania* infection (Tomiotto-Pellissier et al., 2018). The production of microbicide molecules, such as NO and ROS, inflammatory cytokines, and the upregulation of TLRs have been related to the leishmanicidal activity of human and murine macrophages (Faria et al., 2012). However, *Leishmania* has developed several strategies to evade the immune response in the vertebrate host (Atayde et al., 2016; Scott and Novais, 2016). Lipophosphoglycan (LPG) and the glycoprotein metalloprotease gp63 (gp63) are important virulence factors produced by the parasites that act in different mechanisms of host immune response providing an environment permissive for the establishment of *Leishmania* infection (Olivier et al., 2012; Forestier et al., 2014; Barbosa et al., 2018).

Besides macrophages, other cell types can be infected by *Leishmania* and influence the immune response against the parasite (Hurrell et al., 2017; Martínez-López et al., 2018). Our group and others have demonstrated that B-1 cells, a subtype of B lymphocytes, participate in the immune response against *Leishmania* (Arcanjo et al., 2015; Gonzaga et al., 2015; Geraldo et al., 2016). Murine B-1 cells express unusual cell surface markers (CD19^+^CD23^lo^IgM^hi^IgD^lo^CD45^lo^CD11b^+/-^CD43^+/-^ (Baumgarth, 2011; Baumgarth, 2017) and human B-1 cells express CD20^+^CD27^+^CD43^+^ (Griffin and Rothstein, 2012; Quách et al., 2016); they participate in homeostasis and immune response by producing cytokines (mainly IL-10) (O’Garra et al., 1992), and host humoral response (natural and inducible antibodies) (Baumgarth, 2017; Savage et al., 2017). Intriguingly, B-1 lymphocytes express both myeloid and lymphoid commitment transcription factors, which allows them to differentiate into cells with phenotypic, physiological and molecular characteristics of mononuclear phagocytes (Almeida et al., 2001; Popi et al., 2009).

In leishmaniasis, whether B-1 cells participate in resistance or susceptibility depends upon the *Leishmania* species and strain (Gonzaga et al., 2015; Gonzaga et al., 2017). Animals with severe X‐linked immunodeficiency (XID) (due to mutation in Bruton’s tyrosine kinase -Btk) had a significant reduction in peritoneal B-1 cells (Hayakawa et al., 1983; Khan et al., 1995). These animals showed more resistance to chronic infection with *Leishmania infantum* as compared to control (Gonzaga et al., 2015). In contrast, BALB/XID mice were more susceptible to infection by *L. amazonensis* (MHOM/BR/1973/M2269 strain) (Gonzaga et al., 2017). Also, EVs released by B-1 cells led to a reduction in the parasite load in mice experimentally infected by the parasite (Toledo et al., 2020). *In vitro*, stimulation with *L. amazonensis* promastigotes or EVs released by the parasite modulated the cytokines production by B-1 cells (Geraldo et al., 2016; Barbosa et al., 2018). Thus, although the mechanisms are not yet fully understood, it is possible to consider that B-1 cells participate in immunity to *L. amazonensis*. However, the ability of the parasite or their EVs to induce B-1 cell activation and/or differentiation into phagocytes *in vivo* have not yet been evaluated.

Thus, this work investigated the differentiation and activation of B-1 cells after stimulation with *L. amazonensis* or EVs released by the parasites. We evaluated the production of microbicidal molecules, the expression of TLRs, cytokines, and lymphoid and myeloid commitment factors in peritoneal B-1 cells after intraperitoneal stimulation with *L. amazonensis* promastigotes or EVs released by the parasites. Understanding how B-1 cells are activated after stimulation by the parasite and its components can help better to understand the role of B-1 cells in leishmaniasis. This study can unveil the relationship of B-1 cells and *Leishmania* and how they can be related to cutaneous leishmaniasis progression and protozoa parasite-host B-1 communication and activation.

## Material and Methods

### Animals

Six- or 8-week-old female BALB/c mice were purchased from Center for the Development of Experimental Models for Medicine and Biology (CEDEME, Universidade Federal de São Paulo - UNIFESP, São Paulo, SP, Brazil). The mice were housed under pathogen-free conditions, as recommended by the National Council for Control Animal Experimentation (CONCEA) of Brazil. All procedures were approved by the Committee on Ethics of Animal Experiments (CEUA) of UNIFESP, under protocol number 8762030718.

### Parasites

*L. amazonensis* (MHOM/BR/1973/M2269 strain) parasites were kindly provided by Dr. Clara Lúcia Barbieri of UNIFESP, São Paulo, Brazil. The parasites were aseptically cultured as promastigotes at 26°C in 199 medium (Gibco, Life Technologies Brand, Grand Island, NY, United States) supplemented with 4.2 mM sodium bicarbonate, 4.2 mM HEPES, 1 mM adenine (Sigma, St. Louis, MO, United States), 5 µg/ml hemin (bovine type I) (Sigma) plus 10% fetal bovine serum (FBS) (Gibco, Carlsbad, CA, United States). Promastigotes in the stationary growth phase were recovered by centrifugation and used for infection or obtaining EVs.

### Obtaining and Isolating of EVs Released by *L. amazonensis*

*L. amazonensis* promastigotes were cultured *in vitro* until the stationary phase. Parasites were recovered, washed 5 times in phosphate-buffered saline (PBS), and 10^8^ parasites were distributed in each microtube containing 1 ml of medium 199 added with 2% D-dextrose. The parasites were incubated at 26°C for 4 h for EVs releasing (Barbosa et al., 2018). Then, the cultures were centrifuged to remove parasites and EVs were recovered from the supernatants. Afterward, the supernatants were recovered and filtered through 0.45-μm sterile cartridges and submitted to sequential centrifugation at 4°C: 500 g for 10 min, 1,500 g for 10 min, 10,000 g for 10 min, and twice at 100,000 × g for 1 h to pellet parasite EVs. Sorvall WX Ultra Thermo Scientific (rotor T890) was used in the ultracentrifugation steps (Barbosa et al., 2018). The pellets were washed once and then diluted in sterile filtered PBS.

The protein concentrations of isolated EVs were evaluated by using the Micro BCA protein assay kit (Thermo Scientific, Waltham, MA, United States). The concentration of particle number/ml and size distribution of EVs were determined using Nanoparticle Tracking Analysis (NTA) in a Nanosight NS300 instrument (Malvern Instruments Ltd., Malvern, United Kingdom). Samples were diluted 10- to 100-fold in sterile PBS and captured in triplicate for 30 s with the camera level set to 14 using the same threshold along all analyses. The data acquired were examined using the NTA software (version 2.3 build 0017) as previous described (Barbosa et al., 2018).

### Intraperitoneal Inoculation of BALB/c Mice With Parasites or EVs

BALB/c mice were intraperitoneally inoculated with 10^8^ parasites or with 4 µg of EVs (corresponding to 2.39 × 10^6^ particles ± 1.25 × 10^6^). Parasites were cultured as promastigotes until stationary phase. After washing with sterile PBS, parasites were resuspended at a concentration of 10^8^ parasites/500 µl and then intraperitoneally inoculated in mice (adapted from Geraldo et al., 2016). Sterile EVs resuspended in PBS were intraperitoneally inoculated at a concentration of 4 µg/mouse (Barbosa et al., 2018). Animals inoculated with sterile PBS were used as a negative control. 24 or 48 h after inoculation, the animals were euthanized, and peritoneal cavities were washed with ice-cold sterile PBS to collect total peritoneal cells.

### Flow Cytometry

Peritoneal cells were washed with cooled sterile PBS and counted in the Neubauer chamber. The cell suspensions were distributed in 1.5 ml microtubes and diluted up to 100 μl PBS (1 × 10^6^ cell/microtube). The purified rat anti-mouse CD16/CD32 (2.4G2 clone, BD Fc Block™, BD Bioscience, San Jose, CA, USA) was used to block non-antigen-specific binding of immunoglobulins to the Fc receptors. Cell suspensions were incubated with Fc block for 60 min at 4°C. Samples were then incubated with anti-CD19 coupled with allophycocyanin (APC) (clone 1D3, BD) and anti-CD23 coupled with phycoerythrin (PE) (clone B3B4, BD). B-1 cell population was identified as CD23^-^CD19^+^. The following monoclonal antibodies were used to evaluate other cell surface markers: fluorescein-isothiocyanate (FITC)-conjugated anti-mouse CD80 (anti-CD80 FITC, clone 16-10A1, BD), anti-CD86 FITC (clone GL1, BD), anti-CD40 FITC (clone 3/23, BD), anti-F4/80 coupled with peridinin-chlorophyll-protein - PerCP (clone BM8) (BioLegend, San Diego, CA, USA). To evaluate the expression of MHC II, we used anti-MHC II, and B-1 cells were identified with anti-CD19 APC (BD), anti-CD23 FITC (BD). All antibodies were used at 1:100 dilutions. The samples were incubated with fluorochrome-conjugated antibodies for 30 min at 4°C, washed with PBS, resuspended in 500 μl of PBS, and analyzed with BD FACSCalibur flow cytometer (BD Bioscience).

Intracellular NO production was evaluated using the fluorescent reagent 4-amino-5-methylamino-2,7-difluorescein diacetate (DAF2-DA, Sigma) and the production of intracellular ROS was evaluated using the fluorescent reagent H_2_DCFHDA (Sigma). Total peritoneal cells were labeled with anti-CD19 APC and anti-CD23 PE. Then, cells were washed with PBS and followed incubated with 5 µM of DAF2-DA or H_2_DCFHDA for 30 min at 37°C in the dark. Cells were washed with PBS and resuspended with 500 µl of PBS. The data acquisition was performed with the FACSCalibur cytometer (BD), and the results were analyzed with FlowJo software version 10.6.2 (FlowJo, LLC, FlowJo™ Software for Mac, Ashland, OR, USA). The gating strategy is shown in **Supplementary Figure 1**.

### Enrichment of Peritoneal B-1 Cells

Total peritoneal cells from mice stimulated or not with *L. amazonensis* promastigotes or EVs released by the parasite were subjected to centrifugation at 161 × g for 5 min. The pellets containing the cells were resuspended in PBS pH 7.2 supplemented with 0.5% FBS, and 2 mM ethylenediaminetetraacetic acid—EDTA (MACS buffer) and the cells were counted in a Neubauer chamber. For enrichment 90 µl of MACS were added to every 10^7^ total cells. Cell suspensions were incubated with 2 μl of Fc block per 6 × 10^6^ total cells for 60 min at 4°C. Then, cells were washed with sterile PBS, resuspended in MACS, and subjected to negative selection with anti-CD23 microbeads (Miltenyi Biotec, Bergisch Gladbach, Germany) followed by positive selection with anti-CD19 microbeads (Miltenyi Biotec) (CD23^-^ CD19^+^). All procedures were performed according to the manufacturer’s instructions. The purity of the cell suspension was evaluated by flow cytometry using anti-IgM APC (II/41 clone, BD) and anti-CD11b PE (M1/70 clone, BD) cell surface markers. The data were acquired in the FACSCalibur cytometer (BD Bioscience) and analyzed with FlowJo software version 10.6.2 (FlowJo, LLC).

### Quantitative Reverse Transcriptase-Polymerase Chain Reaction

qRT-PCR was employed to analyze the expression of arginase enzyme, Toll-like receptors (TLRs), cytokines, and myeloid and lymphoid commitment transcription factors in B-1 cells from mice intraperitoneally infected with parasites or inoculated with EVs from parasite. All qRT-PCR followed the recommendations of the Minimum Information for Publication of Quantitative Real-time PCR Experiments (MIQE) guidelines (Bustin et al., 2009). First, peritoneal B-1 cells were enriched, and then total RNA was extracted using PureLinkRNA Mini Kit (Ambion; Thermo Fisher Scientific Brand, Grand Island, NY, United States™) following the manufacturer’s recommendations. The quantification of total RNA was performed by evaluating their UV absorption in a spectrophotometer (Nanodrop 2000c, Thermo Fisher). Samples with reading ranges of 1.8–2.0 at 260/280 nm and 260/230 nm were analyzed for their integrity by electrophoresis in 1.5% agarose gels. Then, identical amounts (in μg) of the total RNA samples with high quality and integrity were treated with DNAse (RQ1 RNase-free DNase; Promega, Madison, WI, United States) to remove possible contamination with genomic DNA. The cDNA synthesis was performed with the Proto Script First Strand cDNA Synthesis Kit (New England Biolabs Inc., MA, United States). Treatment with DNAse and cDNA preparation was developed by following the instructions of the manufacturer. All the qRT-PCR were performed with equal amounts of each cDNA using SYBR Green Real-Time PCR Master Mix (Applied Biosystems, Thermo Fisher Scientific) and the apparatus StepOnePlus (Applied Biosystems). The following conditions were used in all qRT-PCR reactions: 1 μl of cDNA, 5.0 μl of SYBR Green Master Mix (Thermo Fisher), and 2.0 μl of each oligonucleotide (1.0 μM); the cycle parameters 10 min at 50°C for enzyme activation, denaturation for 5 min at 95°C, and 40 cycles of 95°C for 30 s and 60°C for 1 min. Internal negative controls were performed by using two approaches: I) reactions were performed with samples without addition of the transcriptase reverse for cDNA preparations; and II) not adding template for qPCR reaction (NTC- no template control). In both cases, no amplifications were detected.

To analyze, first, we checked the quality of the reaction based on the dissociation curves (melting curves), and we always detected the presence of only one peak in each reaction. The baseline was adjusted to two or three cycles before the detection of the fluorescent signal. The cycle threshold (Ct) was defined as follows: I) above the background fluorescence baseline, to avoid the amplification plot crossing the threshold prematurely due to background fluorescence; II) in the log phase of the amplification plot, avoiding the plateau phase; and III) at a position where the log phases of all amplification plots are located in a parallel line.

Before performing the gene expression assays, the efficiencies of all primers used throughout this study were analyzed. The efficiency of primers was evaluated by constructing standard curves with the dilution of the samples. The standard curve always contained four or five different points. The Ct values of each dilution point were determined and used to make the standard curve and to calculate the efficiency of primers using the equation E = 10(-1/slope)-1 (E corresponds to the efficiency, and slope is the slope of the standard curve). Then, we compared the efficiency of primers of target genes and normalizers to validate the best normalizers. The sequences of the primers used for each target gene are shown in the **Supplementary Table 1**. The primers sequences were obtained according PrimerBank (Spandidos et al., 2010).

The relative quantification of genes analyzed was calculated according to the 2^-ΔΔCt^ method (Schmittgen and Livak, 2008). All experiments were performed in triplicates with at least three biological samples. Differences in the relative expression levels of target genes (fold change) were determined by comparing B-1 cells from mice injected with PBS as reference samples (control) with B-1 cells from mice stimulated with parasites or EVs. The gene expression for the reference sample was always adjusted to 1.

### Statistical Analysis

Experiments were carried out with at least three biological replicates. Results from representative experiments are shown. The data are shown as the mean ± standard deviation (SD). Paired one-tailed Student’s t-tests were used to perform statistical analysis. P-values < 0.05 were considered significant. All statistical tests were performed using Graph Pad Prism version 7 for Mac (GraphPad Software, La Jolla, CA, United States).

## Results

### Intraperitoneal Stimulation With *L. amazonensis* Promastigotes or EVs Modulated the Expression of Arginase Enzyme and the Production of NO and ROS in Murine B-1 Cells

**Figure 1:**
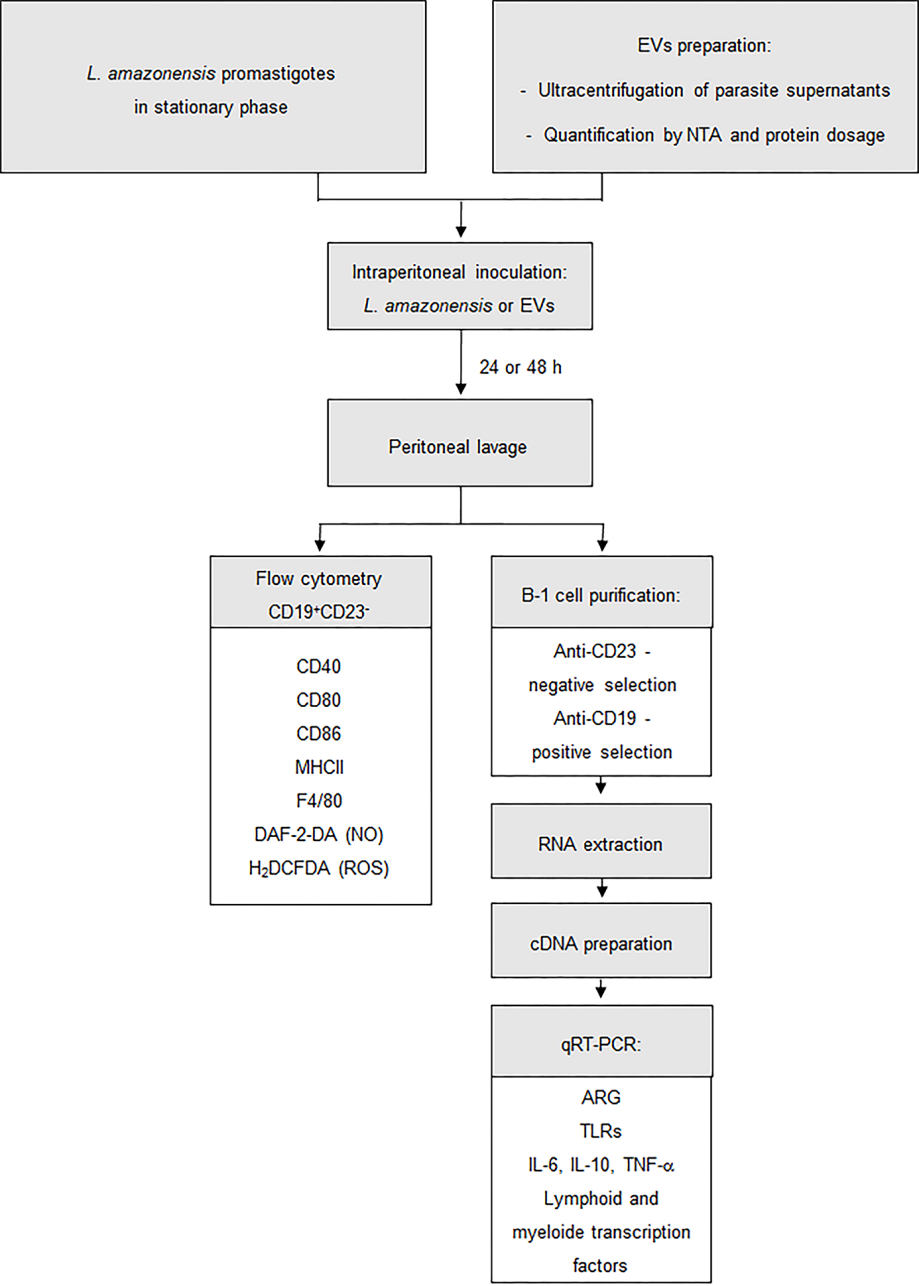
Procedures employed for the enrichment and stimulation assays. Workflow used to develop this study. BALB/c mice were injected intraperitoneally with 1 × 10^8^ *L. amazonensis* promastigotes or 4 ug of EVs (corresponding to 2.39 × 10^6^ particles ± 1.25 × 10^6^). After 24 or 48 h, the total peritoneal cells were collected from mice and then analyzed by flow cytometry or were used to enrich B-1 cells. In flow cytometry, B-1 cells were evaluated for the presence of CD80, CD86, CD40, MHC II, F4/80, NO, and ROS. Enriched B-1 cells were used to analyze the gene expression of arginase, TLRs, cytokines and myeloid and lymphoid transcription factors. *Material and Methods* section explains the details of the methodology employed.

In this work, we evaluated the modulation of peritoneal B-1 cells after intraperitoneal stimulation with *L. amazonensis* promastigotes or EVs released by the parasite. All experiments were performed with *L. amazonensis* stationary promastigotes. The EVs from parasites were obtained as described in the *Material and Methods* section. Isolated EVs exhibited mean diameter sizes of 180.0 ± 12.4 nm (data not shown). No differences in mean size were observed in all EVs purification experiments. In this work, we used the total shed of parasite as previously described (Barbosa et al., 2018). The size of the EVs was larger, 180 nm, than the EVs sizes reported in previous studies (Silverman et al., 2010a; Silverman et al., 2010b; Hassani and Olivier, 2013). However, EVs released from *L. amazonensis* with larger sizes have already been reported (Barbosa et al., 2018; Sauter et al., 2019). As expected, no particles were found in the medium alone (negative control, data not shown). All experiments described in this session are summarized in the workflow in **Figure 1**.

**Figure 2:**
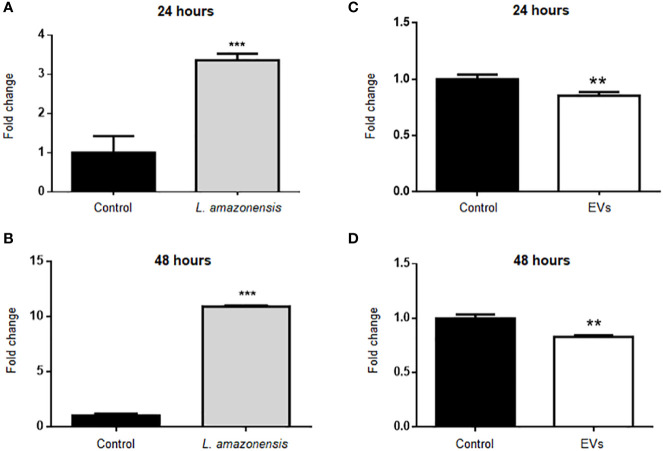
Relative expression of arginase in B-1 cells after intraperitoneal stimulation for 24 or 48 h with *L. amazonensis* promastigotes (left panel) or EVs (right panel). B-1 cells were negatively selected with anti-CD23 microbeads and positively with anti-CD19 microbeads. Animals inoculated with PBS were used as control. The RNA was extracted, the cDNA obtained, and qPCR analyses were performed to verify the arginase enzyme´s gene expression. Infection for **(A)** 24 h and **(B)** 48 h with the parasite, **(C, D)** stimulation for 24 or 48 h with the EVs, respectively. The bars indicate the average of triplicates, and the error bars the standard deviation. The graph is representative of three independent experiments. Test t-student, **P < 0.01; ***P < 0.001.

BALB/c mice were inoculated with parasites (1 × 10^8^/mouse) or EVs (4 µg/mouse, corresponding to 2.39 × 10^6^ particles ± 1.25 × 10^6^) for 24 or 48 h (**Figure 1**). Then, the total peritoneal cells were collected, and B-1 cells were enriched using magnetic selection. The expression of the arginase was evaluated in the enriched B-1 cell population, and a significant increase was observed in B-1 cells of infected animals, compared to cells obtained from uninfected mice at both time points (**Figures 2A, B**). However, the intraperitoneal stimulation with EVs induced a significant decrease in the expression of this enzyme after 24 or 48 h of inoculation (**Figures 2C, D**, respectively).

**Figure 3:**
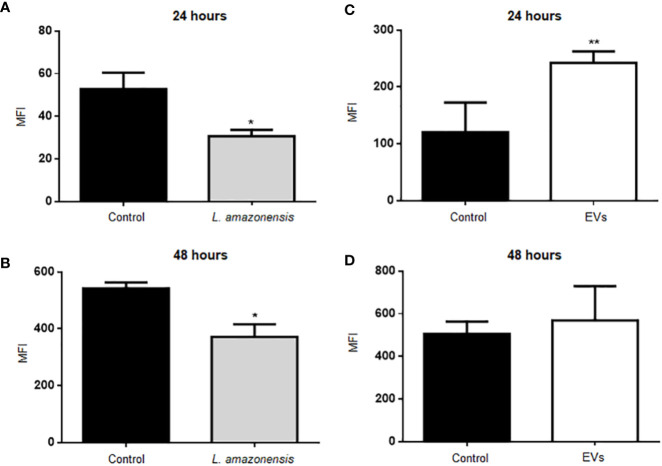
Intracellular NO production in B-1 cells after 24 or 48 h of intraperitoneal inoculation with *L. amazonensis* promastigotes (left panel) or EVs (right panel). **(A)** 24 h of infection with the parasite, **(B)** 48 h of stimulation with the parasite, **(C)** stimulation for 24 h with EVs, and **(D)** 48 h of stimulation with EVs. Total peritoneal cells were collected and labeled with anti-CD19 coupled with APC, anti-CD23 coupled with PE and DAF-2DA. Data acquisition was performed using a FACSCalibur cytometer. Subsequent analyzes were performed using the FlowJo software. The graphs represent the median fluorescence intensity (MFI) for the DAF probe in CD19^+^CD23^-^ cells. The bars represent the mean of the duplicates, and the error bars the standard deviation. The graph is representative of two independent experiments. Test t-student, *P < 0.05; **P < 0.01, comparing stimulated (with EVs or parasites) and unstimulated B-1 cells.

Arginase catalyzes the hydrolysis of L-arginine to L-ornithine and urea. L-arginine is a substrate for inducible nitric oxide synthase (iNOS) to produce NO. Total peritoneal cells were collected and labeled with antibodies anti-CD19 APC and anti-CD23 PE to identify B-1 cells (CD23^-^CD19^+^) and with a fluorescent probe to evaluate NO. BALB/c mice inoculated with PBS were used as the control group. **Figure 3** shows the graphs with the mean values of mean fluorescent intensity (MFI) for intracellular NO in the population of B-1 cells. The results showed a significant decrease in MFI in B-1 cells of animals infected for 24 or 48 h of infection with *L. amazonensis* promastigotes, compared to the uninfected group (**Figures 3A, B**, respectively). B-1 cells from animals stimulated with EVs had an increase in the production of NO after 24 h (**Figure 3C**). However, after 48 h of stimulation (**Figure 3D**) no differences in NO production were observed.

**Figure 4:**
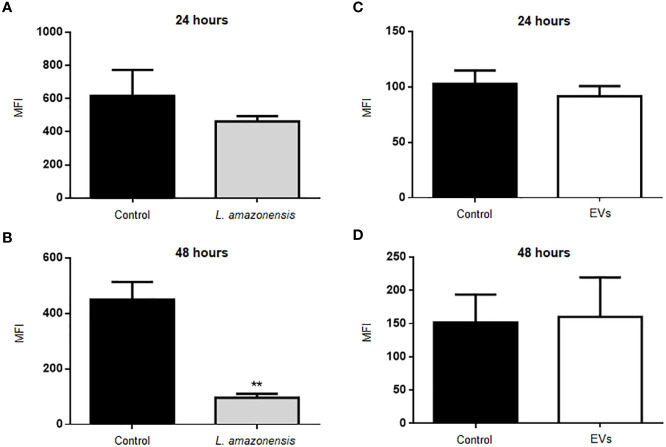
Production of ROS in B-1 cells after 24 or 48 h of stimulation with *L. amazonensis* promastigotes (left panel) or the parasites EVs (right panel). **(A)** infection for 24 h with *L. amazonensis* promastigotes, **(B)** 48 h of stimulation with the parasite, **(C)** 24 h of stimulation with EVs, and **(D)** 48 h of stimulation with EVs. Total peritoneal cells were collected and labeled with anti-CD19 coupled with APC, anti-CD23 coupled with PE, and H_2_DCFHDA. Data acquisition analyzes were performed using a FACSCalibur cytometer, followed by analyzes in FlowJo software. The graphs represent the median fluorescence intensity (MFI) for the H_2_DCFHDA probe in CD19^+^CD23^-^ gating cells. The bars represent the mean of the duplicates, and the error bars the standard deviation. The graph is representative of two independent experiments. Test t-student, **P < 0.01.

We also analyzed the production of ROS in B-1 cells stimulated with parasites and EVs. **Figure 4** shows the graphs with the MFI values of ROS in B-1 cells after 24 or 48 h of the stimulus with the parasite (**Figures 4A, B**, respectively). The results show a significant decrease in MFI in B-1 cells of animals infected for 48 h with *L. amazonensis* promastigotes, compared to the uninfected group (**Figure 4B**). No differences were detected in ROS production in B-1 stimulated for 24 or 48 h with EVs (**Figures 4C, D**). We also evaluated the expression of CD80, CD86, CD40, MHC II, F4/80 in B-1 cells from mice, but no differences were detected in B-1 cells from infected or non-infected animals (data not shown).

Taken together, these results suggest that intraperitoneal stimulation with parasites induced a decrease in the production of microbicidal molecules (NO and ROS) by B-1 cells. On the other hand, intraperitoneal inoculation with EVs stimulated NO production only 24 h after stimulation.

### Expression of TLRs and Cytokines in B-1 Cells of Mice Intraperitoneally Stimulated With *L. amazonensis* Promastigotes or EVs

**Figure 5:**
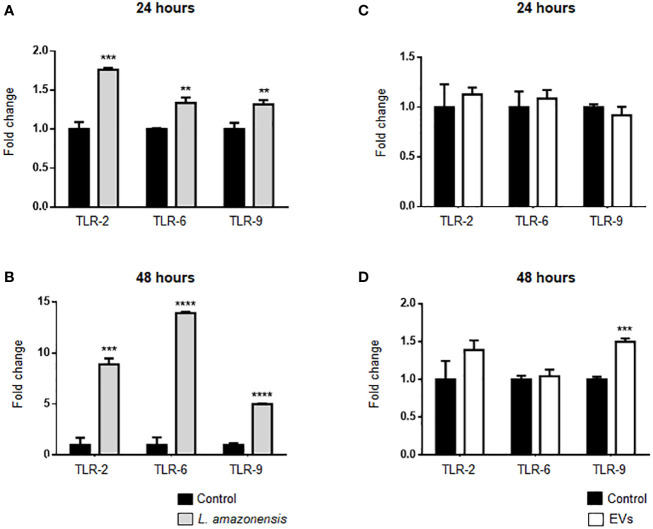
Expression of TLRs in B-1 cells after 24 or 48 h of intraperitoneal stimulation with *L. amazonensis* promastigotes (left panel) or parasite EVs (right panel). **(A, B)** stimulation for 24 and 48 h with parasites, respectively; **(C)** 24 and **(D)** 48 h of stimulation with EVs. B-1 cells were negatively selected with anti-CD23 microbeads and positively selected with anti-CD19 microbeads. Mice intraperitoneally inoculated with PBS were used as control. The RNA was extracted, the cDNA obtained, and qPCR analyses were performed to verify the gene expression of TLR-2, TLR-6, and TLR-9. The bars indicate the average of triplicates, and the error bars the standard deviation. The graph is representative of three independent experiments. Test t-student, **P < 0.01; ***P < 0.001; ****P < 0.0001, comparing stimulated (with EVs or parasites) and unstimulated B-1 cells.

TLRs can interact with *Leishmania* and participate in innate immunity to a pathogen, such as *Leishmania* spp. (Tuon et al., 2010). In addition, *Leishmania* induces upregulation of TLRs in human and murine macrophages (Faria et al., 2012; Chauhan et al., 2017). Herein, TLRs expression was evaluated in B-1 cells of mice intraperitoneally stimulated with parasites or EVs. Enriched B-1 cells obtained 24 or 48 h after i.p. stimulation were used to analyze TLR-2, TLR-6, and TLR-9 expression by qRT-PCR. TLRs showed significantly higher expression levels in B-1 cells of animals stimulated with the parasite for 24 or 48 h, compared to cells from control (**Figures 5A, B**). No changes were detected in TLR-2, TLR-6, and TLR-9 in B-1 cells from animals stimulated for 24 h with parasite EVs (**Figure 5C**). However, after 48 h, a significant increase in the expression of TLR-9 was detected, as compared with control mice (**Figure 5D**). Altogether, our data revealed the differential expression of TLR2, 6, and 9 in B-1 cells recovered from BALB/c mice stimulated intraperitoneally with *L. amazonensis* or their EVs.

**Figure 6:**
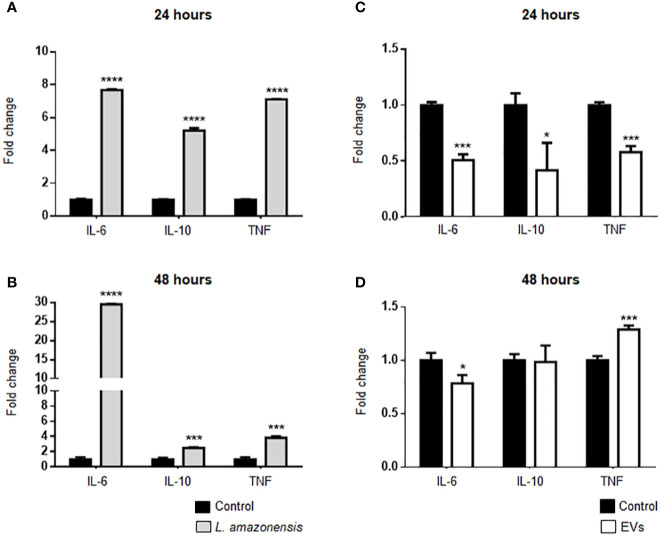
Expression of IL-6, IL-10, and TNF-α in B-1 cells after stimulation for 24 or 48 h with *L. amazonensis* promastigotes (left panel) or EVs (right panel). **(A)** and **(B)** stimulation with the parasites for 24 or 48 h, respectively; **(C)** 24 and **(D)** 48 h of stimulation with EVs. B-1 cells were enriched using negative selection with anti-CD23 microbeads and positive selection with anti-CD19 microbeads. Animals inoculated with PBS were used as control. The RNA was extracted, the cDNA obtained, and qPCR analyses were performed to verify the gene expression of IL-6, IL-10, and TNF-α cytokines. The bars indicate the average of triplicates, and the error bars the standard deviation. The graph is representative of three independent experiments. Test t-student, *P < 0.05; ***P < 0.001; ****P < 0.0001, comparing stimulated (with EVs or parasites) and unstimulated B-1 cells.

The expression of IL-6, IL-10, and TNF-α was evaluated in B-1 cells of BALB/c mice inoculated with *L. amazonensis* promastigotes. B-1 cells showed a significant increase in the expression of these cytokines after the stimulus for 24 and 48 h (**Figures 6A, B**, respectively). It is noteworthy the pronounced increase in IL-6 after 48 h, while the expression of IL-10 and TNF-α had a decrease after 48 h, as compared with 24 h. The peritoneal stimulation with EVs released by the parasites led to a significant decrease in IL-6, IL-10, and TNF-α expression in enriched B-1 cells after 24 h of stimulation (**Figure 6C**). However, after 48 h of EVs intraperitoneal inoculation, we detected in B-1 cells a decrease in the IL-6 expression, no changes in the IL-10 mRNA levels, and an increase in the TNF-α expression (**Figure 6D**). Based on these data, IL-6, IL-10, and TNF-α were differentially expressed in B-1 cells after intraperitoneal stimulation with *L. amazonensis* or EVs. Interestingly, the increase in cytokine expression was observed in the group with the highest expression of TLRs.

### Intraperitoneal Stimulation With *L. amazonensis* Promastigotes or EVs Alters the Expression of Myeloid and Lymphoid Impairment Transcription Factors in Mouse B-1 Cells

**Figure 7:**
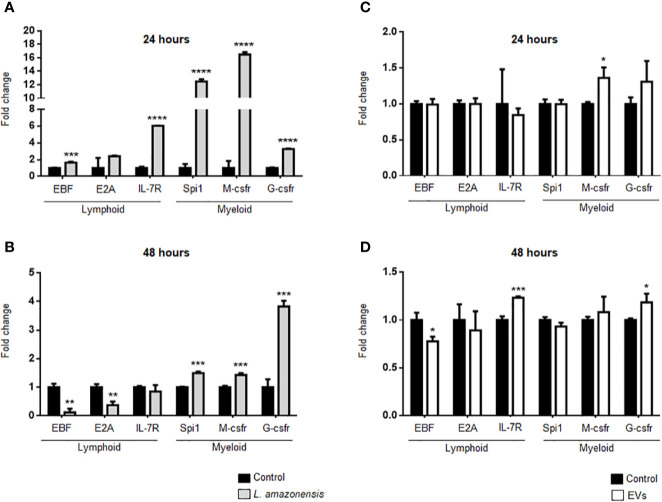
Expression of lymphoid and myeloid restricted transcription factor in B-1 cells after stimulation for 24 or 48 h of intraperitoneal stimulation with *L. amazonensis* promastigotes (left panel) or parasite EVs (right panel). **(A)** infection for 24 h with the parasite, **(B)** stimulation for 48 h with parasite, **(C)** 24 h, and **(D)** 48 h of stimulation with EVs, respectively. B-1 cells were enriched using negative selection with anti-CD23 microbeads and positive selection with anti-CD19 microbeads. Animals inoculated with PBS were used as control. The RNA was extracted, the cDNA obtained, and qPCR analyses were performed to verify the gene expression of EBF, E2A, IL7-r, Spi1, M-csfr, and G-csfr. The bars indicate the average of triplicates and the error bars the standard deviation. The graph is representative of three independent experiments. Test t-student, *P < 0.05; **P < 0.01; ***P < 0.001; ****P < 0.0001, comparing stimulated (with EVs or parasites) and unstimulated B-1 cells.

B-1 cells express both lymphoid and myeloid commitment transcription factors (Popi et al., 2009) and differentiate into phagocytes *in vitro* and *in vivo* (Almeida et al., 2001; Popi et al., 2012). Phagocytes derived from B-1 cells phagocyted *Leishmania* *in vitro* and released cytokines and EVs, which influence the activation of other cells such as macrophages (Arcanjo et al., 2015; Geraldo et al., 2016; Toledo et al., 2020). However, it has not yet been demonstrated whether this differentiation occurs *in vivo* after stimulation with the parasite or their EVs. Thus, we evaluated the effects of intraperitoneal stimulus with *L. amazonensis* or EVs on the differential expression of myeloid or lymphoid commitment transcription factors in the B-1 cells. The expression of myeloid impairment genes (transcription factor PU.1—Spi1, macrophage colony-stimulating factor 1 receptor—M-csfr, and granulocyte colony-stimulating factor 3 receptor—G-csfr) and lymphoid impairment genes (early B cell Factor - EBF, transcription factor 3—E2A, and Interleukin-7 receptor—IL-7r) were analyzed by qRT-PCR. **Figure 7A** shows a significant increase in the expression of all target genes analyzed after 24 h of intraperitoneal stimulation with the parasites, except for the E2A gene that the increase in expression was not significant. However, we observed a more pronounced increase in the expression of myeloid-impaired genes (Spi1, M-csfr, and G-csfr). After 48 h, a decrease in the lymphoid impairment genes (EBF and E2A) was observed, but the myeloid genes remained increased (**Figure 7B**). EVs led to an increase in the expression of M-csfr in B-1 cells after 24 h of stimulation (**Figure 7C**). After 48 h, the EBF levels significantly decreased while the expression of IL -7r and G-csfr showed a significant increase (**Figure 7D**), compared with the control group. Taken together, our results showed that intraperitoneal stimulation with the parasite led to an increase in the myeloid commitment factor, suggesting that B-1 cells differentiated in a myeloid profile. On the other hand, EVs were not efficient in stimulating the differentiation of B-1 cells into phagocytes.

## Discussion

B-1 cells are a group of B cells that can modulate and participate in innate and adaptive host immune responses (Baumgarth, 2011; Novaes e Brito et al., 2019). However, the response of these cells to parasites or EVs released by the parasite are poorly understood. Our group showed that B-1 cells participate in immunity against *L. amazonensis* (Gonzaga et al., 2017). Mice deficient in B-1 cells (XID mice) were more susceptible to chronic infection with *L. amazonensis* as compared to the background BALB/c (Gonzaga et al., 2017). The previous treatment of mouse footpad with EVs released by B-1 cells led to a decrease in the parasite load and the inflammatory marks in mice challenged with *L. amazonensis* promastigotes (Toledo et al., 2020). Herein we analyzed the changes in peritoneal B-1 cells from mice intraperitoneally stimulated with *L. amazonensis* or parasites EVs. A similar model was used to evaluate the population and activation of B-1 cells after intraperitoneal stimulation with LPS, *Francisella tularensis*, heat-killed *Propionibacterium acnes* as well as their components (Cole et al., 2009; Mussalem et al., 2012; Popi et al., 2012; Gambero et al., 2016). This model shows the changes in B-1 cells *in vivo* and minimizes the artifacts that can be induced by *in vitro* stimulation.

Our results showed a decrease in the NO production by B-1 cells from mice inoculated with *L. amazonensis* after 24 or 48 h of protozoa infection. Also, we detected an increase in the expression of arginase in B-1 cells from mice intraperitoneally infected. Arginase is an enzyme that inhibits the production of NO and is involved in the production of ornithine, a precursor to hydroxyproline and polyamines. Classically activated macrophages (M1 macrophages) do not induce arginase activity and, therefore, convert arginine to NO, one of the components used to eliminate intracellular pathogens (Fleming and Mosser, 2011). *Leishmania* parasites can regulate the increase in arginase activity in the host cells (Wilkins-Rodríguez et al., 2020) and the decrease of NO production, leading to the persistence of the pathogen (Gregory and Olivier, 2005; Nandan and Reiner, 2005). Thus, our data demonstrate intraperitoneal *Leishmania* infection induced a reduction in NO production in B-1 cells, which is similar to what has been previously shown for infection macrophages.

On the other hand, the intraperitoneal stimulation with EVs released by *L. amazonensis* induced a different behavior in B-1 cells. After stimulation with EVs, B-1 cells reduced the expression of arginase after 24 and 48 h of stimulation and showed an increase in the NO production 24 h of intraperitoneal stimulation. EVs released by *Leishmania* may contain different parasite molecules, such as LPG and nucleic acids (Srivastava et al., 2013), that can interact with different cell surface receptors such as TLRs (Faria et al., 2012; Chauhan et al., 2017). B-1 cells express TLRs, and the *in vitro* stimulation with several TLRs agonists increased the NO production (Tumurkhuu et al., 2010). Although the *in vitro* stimulation of macrophages with exoproteome or exosomes of *Leishmania* led to an inhibition of NO production (Hassani et al., 2011; Hassani and Olivier, 2013), our results showed that B-1 cells respond differently after *in vivo* stimulation with EVs. In our model, B-1 cells were stimulated with EVs *in vivo*, and their interaction with other cells and cytokines most probably occurred. A better understanding of the activation mechanisms of B-1 cells and their interaction with parasites, their components, and the immune system can lead to uncover their role in leishmaniasis.

ROS can also participate in the elimination of intracellular pathogens (Nüsse, 2011). In mitochondria, ROS may be produced by NADPH oxidases that reduce oxygen using NADPH as the electron donor to produce superoxide anion (Babior, 1999). Superoxide radicals can be converted to hydrogen peroxide (H_2_O_2_), which is toxic to many pathogens (Ganguli et al., 2019). Non-activated macrophages infected *in vitro* with *L. amazonensis* showed low or insufficient ROS production to eliminate *L. amazonensis* (Scott and Novais, 2016). Our results showed no differences in the ROS production by B-1 cells after 24 h of the intraperitoneal infection with *L. amazonensis.* However, after 48 h of intraperitoneal stimulation with the parasite, a significant reduction in the ROS production was detected in B-1 cells. The stimulation with EVs had no changes in the detection of ROS by B-1 cells. Thus, the intraperitoneal stimulation with the parasite induced a decrease in the production of two important microbicidal molecules by B-1 cells but the stimulation with EVs increased the NO production. B-1 cells participate in immunity against *L. amazonensis* since the presence of these cells or their EVs induce resistance to infection (Gonzaga et al., 2017; Toledo et al., 2020). How these cells act in response to parasites or their components is essential to understand the mechanisms involved in this resistance. Thus, probably B-1 cells participate in immunity to *L. amazonensis* by different mechanism.

Regarding the expression of cell surface receptors, the change in the expression of TLRs by B-1 cells from intraperitoneally infected animals was notable. Toll-type receptors (TLRs) are transmembrane glycoproteins with an important role in the innate and adaptive immune response (Tuon et al., 2010; Gurung and Kanneganti, 2015; Fitzgerald and Kagan, 2020). They are found in macrophages, dendritic cells, NK cells, T and B lymphocytes, including B-1 cells (Arancibia et al., 2007; Popi et al., 2016). TLR-2 and TLR-4 have been considered important in the development of the inflammatory response and pathology in several infectious diseases, such as tuberculosis, malaria, and toxoplasmosis (Mukherjee et al., 2016). Several studies have shown the recognition of *Leishmania* spp. by different TLRs (Becker et al., 2003; de Veer et al., 2003) and an important correlation in the protective responses against *Leishmania* parasites with high expression of TLR-2, -4, and -9 in parasite infections were identified (Martínez-Salazar et al., 2008; Faria et al., 2012). A higher expression of TLR-2 was identified in monocytes from patients with cutaneous leishmaniasis (CL) (Carneiro et al., 2016; Polari et al., 2019). The frequency of monocytes expressing TLR-9 was related to lesion size (Vieira et al., 2013) and granuloma formation (Tuon et al., 2010) in patients with CL. Besides parasites, recently, an interaction between TLR9 and EVs released by *L. amazonensis* amastigotes had been reported in macrophages stimulated *in vitro* (Sauter et al., 2019). Our study demonstrated an increase in the expression of TLR-2, -6, and -9 in B-1 cells after intraperitoneal stimulation for 24 and 48 h with *L. amazonensis* promastigotes. However, intraperitoneal stimulation with EVs induced an increase in the expression of TLR-9 in B-1 cells after 48 h. The presence of nucleic acids in parasite EVs can explain the increase in TLR-9 expression in B-1 cells. Analysis of the content present in *L. amazonensis* EVs are ongoing in our laboratory.

IL-10, IL-6, and TNF-α play an important role in *Leishmania* infection. IL-10 is produced by many cell types and plays a regulatory role in the immune response, by inhibiting the production of inflammatory mediators, and the activation of monocytes and macrophages (Sabat, 2010). *L. amazonensis* stimulates the IL-10 expression making a permissive environment that favors the intracellular parasite survival and growth (Castellano et al., 2015). IL-6 is a pleiotropic cytokine produced by several cell types. Their biological effects include differentiation of macrophages, participation in Th2 polarization, and inhibition of Th1 differentiation leading to a non-protective immune response against *Leishmania* infections (Dienz and Rincon, 2009; Velazquez-Salinas et al., 2019). On the other hand, TNF-α is a pro-inflammatory cytokine that is related to active disease (Nateghi et al., 2016). An increase in cytokine expression has also been identified in B-1 cells from intraperitoneally infected animals. The mRNA levels of IL-6, IL-10, and TNF-α had increased in B-1 cells from animals stimulated with the parasite for 24 and 48 h, compared to cells from control animals (inoculated with PBS). It is notable that after 48 h there was a very pronounced increase in IL-6 expression. Our group demonstrated that the interaction between *Leishmania* and B-1 cells *in vitro* and *in vivo* induced a significant increase in cytokine expression (Geraldo et al., 2016; Barbosa et al., 2018). The activation of TLRs can activate different cell signaling pathways leading to an increase in cytokine production (Fitzgerald and Kagan, 2020). In our model, we observed an increase in the expression of TLR-2, -6, and -9 in B-1 cells from mice intraperitoneally infected, which can be related with the increase in the expression of cytokines in these cells.

On the other hand, after 24 h of stimulation with EVs released by *L. amazonensis* promastigotes, a significant decrease in the cytokine expression were detected in B-1 cells. After 48 h, there was a significant decrease in IL-6 expression, no changes in IL-10 mRNA levels, and a significant increase in the TNF-α expression. No differences in the TLRs were detected in B-1 cells stimulated with EVs except for TLR-9 after 48 h of stimulation. TLR-9 is located intracellularly and activated by DNA sequences. Some experimental models have related the activation of this receptor to the production of TNF-α (Lim et al., 2006; Ivory et al., 2008; Grassin-Delyle et al., 2020). Our results showed an increase in the expression of TLR-9 and TNF-α in B-1 cells from animals intraperitoneally inoculated with EVs. Although additional studies need to be carried out, the correlation in the expression of these two genes cannot be ruled out.

Molecular studies have shown that B-1 cells express mRNA of genes for the lymphoid and myeloid compromising profile (Popi et al., 2009). In our experimental model, the intraperitoneal stimulation with *L. amazonensis* promastigotes for 24 h led to a significant increase in the expression of myeloid and lymphoid-impaired genes in B-1 cells. However, the increase in the expression of myeloid genes was significantly higher compared to the lymphoid genes in these cells. After 48 h of infection, we detected a decrease in the expression of lymphoid genes, while myeloid maintained their higher expression. A similar mRNA expression profile was observed in B-1 cells from mice intraperitoneally stimulated with the *P. acnes* and the soluble polysaccharide fraction of the bacterium (Mussalem et al., 2012) and was related with the early induction of B-1 cells into phagocyte-like cells. The intraperitoneal treatment with LPS also induced differentiation of B-1 cells into phagocytes (Popi et al., 2012). Our group and others have shown that phagocytes derived from B-1 cells can phagocytize *Leishmania* promastigotes more efficiently than macrophages (Arcanjo et al., 2015; Geraldo et al., 2016). In addition, a recent work showed that B-1 cells stimulated *in vitro* with the *Leishmania* parasites secreted more EVs and displayed a morphological change with macrophage characteristics (Toledo et al., 2020). Herein we showed that the intraperitoneal stimulation with *L. amazonensis* can induce the *in vivo* differentiation of B-1 cells into phagocyte-like cells by increasing the expression of myeloid compromising factors.

The intraperitoneal stimulation with EVs induced minimal changes in the expression of lymphoid and myeloid transcription factors. After 24 h, there was a significant increase in the M-csfr gene (myeloid). However, after 48 h, the expression of EBF decreased and IL-7r increase (both lymphoid genes). The myeloid gene G-csfr showed an increase in expression after 48 h of intraperitoneal stimulation with *Leishmania* derived EVs. Thus, although intraperitoneal stimulation with parasites induced a change in gene expression for a myeloid profile in B-1 cells, the inoculation of EVs was not as efficient in inducing a pronounced increase in the expression of myeloid compromising genes. Additional studies to better assess the involvement of B-1 cells for the lymphoid/myeloid profile are underway by our group.

Several studies have shown the role of B-1 lymphocytes in the immune response to different pathogens (Baumgarth, 2011; Baumgarth, 2017; Novaes e Brito et al., 2019). Collectively our results showed that the intraperitoneal inoculation of *L. amazonensis* promastigotes or EVs released by the parasites differentially modulated B-1 cells, altering the production of microbicidal molecules, expression of TLRs, cytokines, or lymphoid/myeloid compromising factors. When combined with other studies, our ﬁndings offer a better understanding of the B-1 cells after intraperitoneal stimulation. B-1 cells can produce cytokines, differentiate into phagocytes and modulate innate and adaptative immune response. Since B-1 cells are able to differentiate into phagocytes, part of the phagocyte population presents during *Leishmania* infection can be derived from B-1 cells and can act differently than macrophages to promote immune response and to eliminate the parasite. This work showed by molecular evidences that this differentiation can occurs *in vivo*. Besides parasites, our study with EVs and their role in the immunobiology of infectious diseases has brought relevant and important information for the knowledge on the pathogenesis of diseases, as well as the pathogen-host relationship.

## Data Availability Statement

The raw data supporting the conclusions of this article will be made available by the authors, without undue reservation.

## Ethics Statement

The animal study was reviewed and approved by Committee on Ethics of Animal Experiments (CEUA) UNIFESP, protocol number 8762030718.

## Author Contributions

NR, TD, and CC performed the experiments. MT and VO assisted with real-time PCR experiments. AP, AT, and PX helped with data analysis and discussion of results. NR and PX wrote the manuscript. PX conceived and designed the study, and sought funds for this project. All authors contributed to the article and approved the submitted version.

## Funding

This work was supported by the Fundação de Amparo à Pesquisa do Estado de São Paulo (FAPESP) (Grant Numbers 2016/17245-4,and 2019/21614-3), and by scholarships from FAPESP (technical training—2018/06597-2), Conselho Nacional de Desenvolvimento Científico e Tecnológico (CNPq), and Coordenação de Aperfeiçoamento de Pessoal de Nível Superior (CAPES).

## Conflict of Interest

The authors declare that the research was conducted in the absence of any commercial or financial relationships that could be construed as a potential conflict of interest.

## Supplementary Material

Supplementary Figure 1 (S1). Gating strategy for flow cytometry. The lymphocyte gate was analyzed for their expression of CD19, and CD23 cell surface markers. CD19^+^CD23^-^ cells were gated and the expression of CD80, CD86, CD40, F4/80, MHC II, NO (labeled with DAF-2DA) and ROS (labeled with H_2_DCFDA) analyzed. This figure is representative of labeling with DAF-2DA.
