# Supplementary figures and images for: Leishmania amazonensis Promastigotes or Extracellular Vesicles Modulate B-1 Cell Activation and Differentiation
Source: Front Cell Infect Microbiol. 2020 Oct 30;10:573813. doi: 10.3389/fcimb.2020.573813 (PMC7662559; doi:10.3389/fcimb.2020.573813)

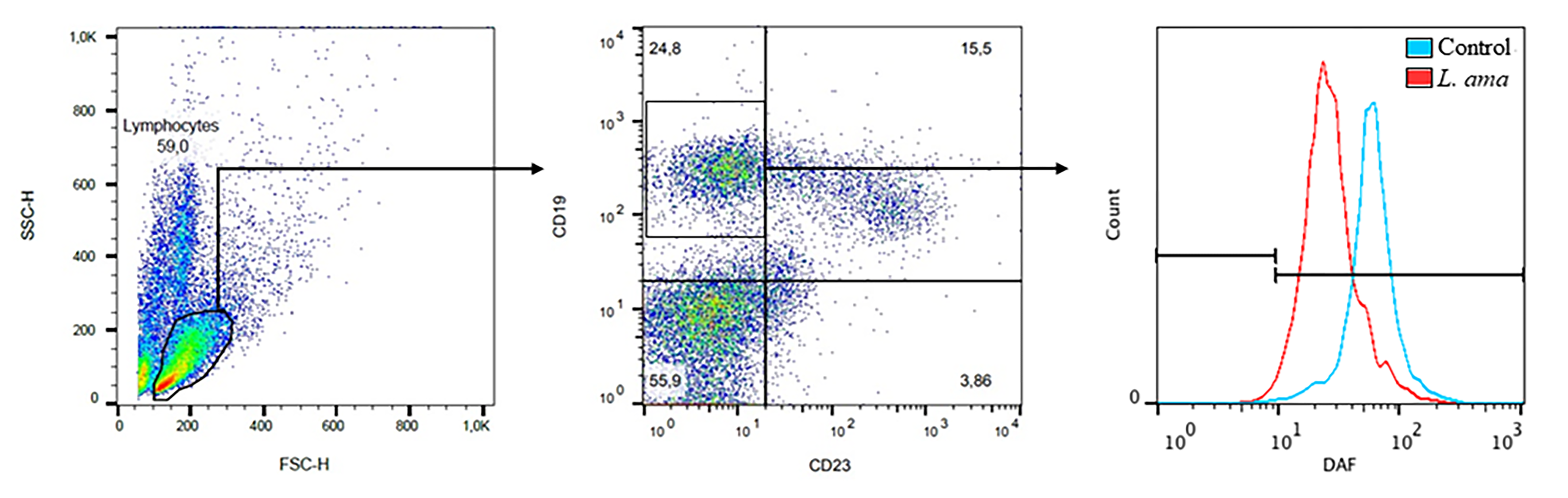

Supplement: Supplementary Figure 1 — (S1). Gating strategy for flow cytometry. The lymphocyte gate was analyzed for their expression of CD19, and CD23 cell surface markers. CD19+CD23- cells were gated and the expression of CD80, CD86, CD40, F4/80, MHC II, NO (labeled with DAF-2DA) and ROS (labeled with H2DCFDA) analyzed. This figure is representative of labeling with DAF-2DA. [file Image_1.tif]
