# Supplementary material for: Leishmania amazonensis Promastigotes or Extracellular Vesicles Modulate B-1 Cell Activation and Differentiation
Source: Front Cell Infect Microbiol. 2020 Oct 30;10:573813. doi: 10.3389/fcimb.2020.573813 (PMC7662559; doi:10.3389/fcimb.2020.573813)
Supplement: Supplementary file 2 [file Table_1.docx]

**Supplementary Table 1**: Sequences of primers used for qRT-PCR reaction

| **Genes** | **Foward**  (5’ – 3’) | **Reverse**  (5’ – 3’) |
| --- | --- | --- |
| IL-7Rα^*^ | AGCTGTTTCTGGAGAAAGTGG | AACGACTTTCAGGTCAGAGGG |
| EBF^*^ | GGTGGAAGTCACACTGTCGTAC | GTAACCTCTGGAAGCCGTAGTC |
| E2A^*^ | CCATGCTAGGTGACGGCTCTTC | GCGAGCCATTAACCTCAGATCC |
| M-CSFR^*^ | TGTCATCGAGCCTAGTGGC | CGGGAGATTCAGGGTCCAAG |
| Spi1^*^ | AGTTCAGGACTTCAGGGAA | TTGCTGCTGTAAGCAGAGGT |
| G-CSFR (csf3r)^*^ | CTCAAACCTATCCTGCCTCATG | TCCAGGCAGAGATGAGCGAATG |
| IL-6^*^ | TAGTCCTTCCTACCCCAATTTCC | TTGGTCCTTAGCCACTCCTTC |
| IL-10^*^ | GCTCTTACTGACTGGCATGAG | CGCAGCTCTAGGAGCATGTG |
| IL-12^*^ | GGAAGCACGGCAGCAGAATA | AACTTGAGGGAGAAGTAGGAATGG |
| TNF-α^*^ | GAAAAGCAAGCAGCCAACCA3 | CGGATCATGCTTTCTGTGCTC |
| ARG^*^ | CTCCAAGCCAAAGTCCTTAGAG | GATGTCCCTAATGACAGCTCCT |
| TLR-2^*^ | CTCTTCAGCAAACGCTGTTCT | GGCGTCTCCCTCTATTGTATTG |
| TLR-6^*^ | AGCCAAGACAGAAAACCCATC | GGGGTCATGCTTCCGACTAT |
| TLR-9^*^ | ATGGTTCTCCGTCGAAGGACT | GAGGCTTCAGCTCACAGGG |
| ARBP | AGCTGAAGCAAAGGAAGAGTCGGA | ACTTGGTTGCTTTGGCGGGATTAG |
| GADPH | AAATGGTGAAGGTCGGTGTG | TGAAGGGGTCGTTGATGG |

^*^Sequences from PrimerBank (Spandidos et al., 2010).
